# A Plant Bioreactor for the Synthesis of Carbon Nanotube Bionic Nanocomposites

**DOI:** 10.3389/fbioe.2020.560349

**Published:** 2020-11-05

**Authors:** Giulia Magnabosco, Maria F. Pantano, Stefania Rapino, Matteo Di Giosia, Francesco Valle, Ludovic Taxis, Francesca Sparla, Giuseppe Falini, Nicola M. Pugno, Matteo Calvaresi

**Affiliations:** ^1^Dipartimento di Chimica “Giacomo Ciamician,” Alma mater Studiorum—Università di Bologna, Bologna, Italy; ^2^Laboratory of Bio-Inspired, Bionic, Nano, Meta Materials and Mechanics, Department of Civil, Environmental and Mechanical Engineering, University of Trento, Trento, Italy; ^3^Istituto per lo Studio dei Materiali Nanostrutturati (CNR-ISMN), Consiglio Nazionale delle Ricerche, Bologna, Italy; ^4^Department of Pharmacy and Biotechnology, Alma mater Studiorum–Università di Bologna, Bologna, Italy; ^5^School of Engineering and Materials Science, Queen Mary University of London, London, United Kingdom

**Keywords:** bionic synthesis, nanobio composite, nanobio interactions, carbon nanotubes, plant nanobioreactor

## Abstract

Bionic composites are an emerging class of materials produced exploiting living organisms as reactors to include synthetic functional materials in their native and highly performing structures. In this work, single wall carboxylated carbon nanotubes (SWCNT-COOH) were incorporated within the roots of living plants of *Arabidopsis thaliana*. This biogenic synthetic route produced a bionic composite material made of root components and SWCNT-COOH. The synthesis was possible exploiting the transport processes existing in the plant roots. Scanning electrochemical microscopy (SECM) measurements showed that SWCNT-COOH entered the vascular bundles of *A. thaliana* roots localizing within xylem vessels. SWCNT-COOH preserved their electrical properties when embedded inside the root matrix, both at a microscopic level and a macroscopic level, and did not significantly affect the mechanical properties of *A. thaliana* roots.

## Introduction

Nanocomposite materials are attracting growing attention in current research for their promising applications in relevant fields such as catalysis (Walsh et al., 2011), medicine (Magnabosco et al., 2015) and sensing (Zhang et al., 2010). Currently, their fabrication routes consist mainly in chemical- and physical- based techniques. The conditions used in these synthetic protocols are usually harsh and employ elevated temperatures and pressures, organic solvents and toxic and expensive chemicals. Living organisms can offer great assistance to chemists to synthesize new materials (Valentini et al., 2016; Ashfaq et al., 2018; Nguyen et al., 2018; Magnabosco et al., 2019; Pugno and Valentini, 2019). After all, living organisms are essentially “nano-factories.” The synthesis of nanomaterials/nanocomposites using biology can be far superior to other technological methods in terms of cost and their eco-friendly nature. Biological entities can control precisely the composition and morphology of nanocomposites while working in physiological conditions, close to neutral pH and at room temperature. The exploitation of living organisms to direct the syntheses of nanocomposites is extremely attractive for two main reasons: (i) the production of the materials takes place in conditions milder than those used in traditional materials-processing techniques; (ii) the reduction of energy inputs and chemicals required to produce the nanocomposites makes bio-enabled syntheses inherently “green” processes. The latter is particularly relevant for *in vitro* or *in vivo* applications since the reagents that are used in chemical syntheses are often toxic and may remain as contaminants in the final product.

Composites made with carbon nanomaterials (CNMs) have been extensively studied to obtain new materials that exploit their chemical and physical proprieties (Calvaresi et al., 2013; Giosia et al., 2016). In particular, carbon nanotubes (CNTs) are one-dimensional (1-D) carbon nanoparticles having excellent electrical, thermal and mechanical proprieties. It is known that CNTs may interact with proteins and living organisms (Long et al., 2012; Calvaresi and Zerbetto, 2013; Wang et al., 2013; Dewi et al., 2015; Lukhele et al., 2015; Thakkar et al., 2016; Di Giosia et al., 2019, 2020); however, most of the work is limited to the study of their toxicity and does not explore their use as an additive to obtain new materials. The inclusion of CNM into plants, their organelles, and organelle components has the potential to enhance their functions (Faltermeier et al., 2014; Wong et al., 2016). For example, CNTs incorporated into isolated thylacoid enhance their maximum electron transport rates (Dewi et al., 2015). A pioneering study by Girardo et al. showed how the direct injection of functionalized CNT into *A. thaliana* leaves allows to prepare a bionic leaf which can be used as a NO sensor (Faltermeier et al., 2014).

Incorporation of nanomaterials inside a whole living plant to create innovative nanocomposites simply by growing the plants in a medium containing the additive is a big challenge.

In this paper, we demonstrated for the first time the possibility to incorporate single wall carboxylated carbon nanotubes (SWCNT-COOH) into the roots of living plants of *Arabidopsis thaliana* to obtain a bionic composite made of root material and SWCNT-COOH, by exploiting the transport properties of *A. thaliana* roots. The bionic incorporation of nanomaterials inside roots directly by using the plant as a “nanoreactor” represents a pioneering approach able to overcome the limitations of laboratory synthesis.

## Materials and Methods

### Water Dispersion of SWCNT-COOH

SWCNT-COOH were purchased by Cheap Tubes (Short COOH Functionalized Single Walled-Double Walled Carbon Nanotubes 1–4 nm). Water dispersions of SWCNT-COOH were obtained by ultrasonication using a probe tip sonicator (Misonix XL2020; 500 W, 40% power) cooling the solution with ice bath, in pre-milliQ water at the concentration of 0.1 mg/mL for 20 min. Lower concentrations were obtained by dilution.

### Plant Growing Methods

*Germination*. The percentage of germination of *A. thaliana* (ecotype Columbia) seeds was calculated by counting seedlings at the emerging of the first true leaves (about 2 weeks after sowing). Seeds were sterilized with chlorine fumes for 4 h and then transferred into square plates (10 × 10 cm^2^ Petri dishes with grid) containing half-strength Murashige-Skoog medium (½MS medium; Micropoli, Milan, Italy), 8 g/L agar and SWCNT-COOH at different concentrations (0.1 mg/mL, 10 μg/mL, and 1 μg/mL). To avoid SWCNT-COOH precipitation, different concentrations of SWCNT-COOH were sterilized at 121°C for 20 min in presence of water-dispersed agar. A stock solution of ½MS medium 100× was sterilized separately, cooled to 40°C and then mixed with SWCNT-COOH/agar solutions to reach the correct final concentration. Four-day cold stratification (the seeds were subjected to both cold, 4°C, and moist conditions. *Arabidopsis thaliana* seeds require these conditions before germination) in the dark was applied prior to transfer seeds into growth chamber. Plants were cultured at 22°C, with cycle of 12-h light/12-h dark, and illuminated with a photosynthetic photon flux density of 110 μmol m^–2^ s^–1^.

*Hydroponic Culture*. Plants were grown in ½MS medium (not supplemented with agar) in the condition described above. Seedholders were filled with 6.5 g/L sterile agarose and containing one seed each. Plants were grown for 28 days allowing the development of a long radical apparatus. After 28 days the plants were transferred to water solution supplemented with dispersed SWCNT-COOH at a concentration of 10 μg/mL, 1 μg/mL, 0.1 μg/mL, and pure water. Plants were kept for a further 6 days under the conditions mentioned above.

### Microscopic Characterization of the Plant Roots

*Scanning Electron Microscopy*. SEM images were collected on uncoated samples using a Phenom G2 and a Zeiss (LEO) 1530VP. The roots were fixed with glutaraldehyde 2.5% in PBS and then air dried. To obtain sections, roots were embedded into polydimethylsiloxane (PDMS) and cut. The root was sliced at the level of the mid-elongation zone, in between the apex and the stem.

*Atomic Force Microscopy*. Substrate surfaces used for AFM imaging were native Silicon Oxide (SiOx) functionalized with 3-Aminopropyltriethoxysilane (APTES). Briefly, the SiOx pieces were first cleaned by the sequential sonication in Acetone, Isopropyl alcohol, and milliQ water; then they were exposed to an oxygen plasma for 5 min. The fragments were then closed in a desiccator containing 10 ul APTES and 10 ul triethanolamine under a mild vacuum for 30 min; 5 ul of CNT solution was spotted onto the APTES-SiOx and dried. Images were performed using a Multimode VIII AFM (Bruker) equipped with a Nanoscope V controller and operated in Peakforce tapping mode; NSG01 probes (NT-MDT) were used. Images were processed by Gwyddion.

### Electrochemical Measurements

Scanning electrochemical microscopy (SECM) measurements were performed using an experimental setup coupling a 910B SECM (CH Instruments) with a Nikon ECLIPSE Ti inverted optical microscope. The stepper motors and the piezoelectric components of the 910B CHI instrument for the microelectrode displacement were removed from the original stage and mounted on the plate of the inverted microscope. SECM measurements were performed on roots cross-section using ferrocenemethanol 1 mM as redox mediator in PBS. A 10 μm platinum disk electrode with an RG = 10 was used as working electrode, an Ag/AgCl (3 M KCl) and a platinum wire acted as reference and counter electrodes, respectively. Data analysis and approach curve fitting were performed using the software MIRA. Conductance was measured with a digimaster tester (DM39A) along 1 cm of root.

### Tensile Tests

Tensile tests were carried out on fresh roots, using the Nanotensile Tester T150 UTM by Agilent. Root samples with typical gage length of 13 mm were tested at room temperature and at a strain rate of 0.001 s^–1^. The measurements were repeated for 6 different samples on control and SWCNT-COOH treated roots.

## Results

### Germination Studies in Presence of SWCNT-COOH

Germination studies were performed to evaluate the *A. thaliana* seeds ability to germinate in presence of SWCNT-COOH (Supplementary Figures S1, S2) and to incorporate the SWCNT-COOH during this growth stage. The germination of *A. thaliana* was not affected by the presence of SWCNT-COOH in the examined interval of concentrations (1–100 μg/mL). SWCNT-COOH treated samples appear very similar to the control, suggesting that no incorporation of the SWCNT-COOH happened. SEM image analysis of the SWCNT-COOH/agar matrix showed that SWCNT-COOH are strongly embedded within the agar matrix (Supplementary Figure S3).

### Hydroponic Culture in the Presence of SWCNT-COOH

**FIGURE 1:**
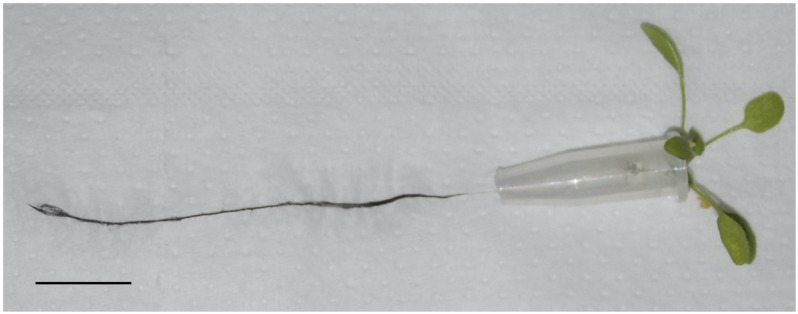
*Arabidopsis thaliana* root treated with SWCNT-COOH at a concentration of a 1 μg/mL after 6 days. Scale bar 5 mm.

Twenty-eight day old plants were transferred in dispersions of SWCNT-COOH in deionized water at different concentrations (10 μg/mL, 1 μg/mL, 0.1 μg/mL, pure water) for 6 days. After 6 days, roots changed coloration and some plants died (see Supplementary Figures S4, S5). All the samples treated with SWCNT-COOH showed an increased mortality compared to the control (Supplementary Figure S5). The effect of SWCNT-COOH on *A. thaliana* vitality was concentration dependent. At the maximum concentration (10 μg/mL) and at the lowest concentration (0.1 μg/mL), a minimum toxic effect was observed. At the intermediate concentration of SWCNT-COOH (1 μg/mL), a strong toxic effect was observed. As can be seen in Figure 1, after 6 days of SWCNT-COOH treatment, the aspect of roots of the survived plants changed visibly, became black and remained black also after repeated washing.

UV-vis analysis (Supplementary Figure S6) of the three solutions (10 μg/mL, 1 μg/mL, and 0.1 μg/mL) showed a signal of the SWCNT-COOH absorbance proportional to the SWCNT-COOH concentration.

Analysis of the AFM images and relative height profile analysis revealed the dispersion state of the SWCNTs-COOH in deionized water at the different concentrations (Supplementary Figure S7). At the maximum concentration (10 μg/mL) SWCNTs-COOH are present in form of bundles of different sizes, while at the intermediate concentration (1 μg/mL) the SWCNT-COOH are monodispersed. At the lowest concentration, no clear SWCNT-COOH signal was detected, also at higher magnification, demonstrating the very low amount of SWCNT-COOH present in solution at this concentration (0.1 μg/mL), as confirmed also by the UV-vis spectra (Supplementary Figure S6).

### Microscopic Characterization of the SWCNT-COOH/Plant Roots Nanocomposite

**FIGURE 2:**
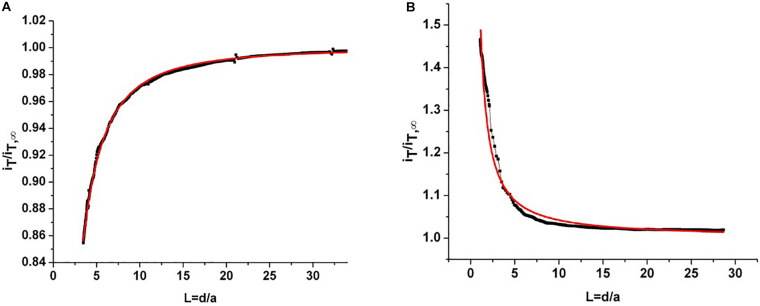
**(A)** Experimental approach curve on non-treated root (black line) and fitted curve using a negative feedback model for insulating substrates (red line). **(B)** Experimental approach curve on root treated with SWCNT-COOH (black line) and fitted curve using a positive feedback model for conductor substrates (red line). iT/iT∞ is the current recorded at the probe divided by the current recorded at the probe in the bulk and *L* = *d/a* is given by the probe-sample separation divided by the ultramicroelectrode (probe) active radius.

**FIGURE 3:**
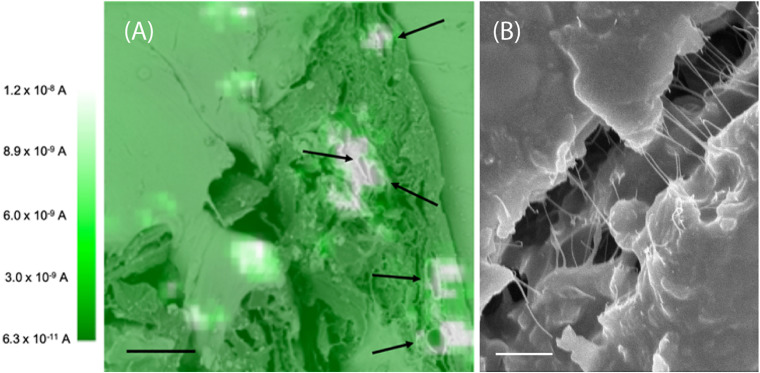
**(A)** SECM image of an *A. thaliana* root section overlapped with SEM image. The white spots are conductive regions due to the presence of SWCNT-COOH. The black arrows indicate the plant vessels. These images are representative of the entire population of samples. Scale bar of 10 μm. The SECM image was performed in 1 mM ferrocenemethanol in PBS, using a 10 μm Pt working electrode, *E* = 0.5 V versus Ag/AgCl (3 M KCl). **(B)** Scanning electron microscopy image of a SWCNT-COOH rich region of the *A. thaliana* root. Scale bar of 1 μm.

The root slices were analyzed with scanning electron microscopy (SEM) and scanning electrochemical microscopy (SECM). The SECM technique enables spatially resolved imaging of SWCNT-COOH avoiding the use of probes and stimuli that may destroy the biological tissue, taking advantage of SWCNT-COOH electrical proprieties (Rapino et al., 2010, 2014; Lin et al., 2018). The presence of SWCNT-COOH was confirmed by SECM approach curves on the roots of treated and non-treated samples (Figure 2A). In fact, the approach curves on non-treated roots showed a typical insulating behavior described by the negative feedback model for insulating substrate, where a decrease of the faradic current is recorded when the sample is approached as an effect of the hindered diffusion of the redox mediator to the probe due to the physical presence of the insulating sample (Lin et al., 2018). On the contrary, the SWCNT-COOH treated samples act as electric conductors, described by the positive feedback model for conductive substrates, as the nanotubes present in the roots are able to regenerate the redox mediator in its pristine form by quickly exchanging electrons with it (Figure 2B) (Rapino et al., 2010). By overlapping a SECM map and a SEM image (Figure 3A) of the same region, it can be observed that the most conductive regions correspond to root vascular bundle. The incorporation of the SWCNT-COOH lowers the resistivity of the roots. In fact, non-treated root showed a resistance of 5.9 MΩ while a treated one has a resistance of 1.0 MΩ, measured with a tester along 1 cm of root. The SEM image of a SWCNT-COOH rich region into the *A. thaliana* root (Figure 3B) shows that SWCNT-COOH bundles are incorporated within the root matrix.

### Mechanical Characterization of the SWCNT-COOH/Plant Roots Nanocomposite

**FIGURE 4:**
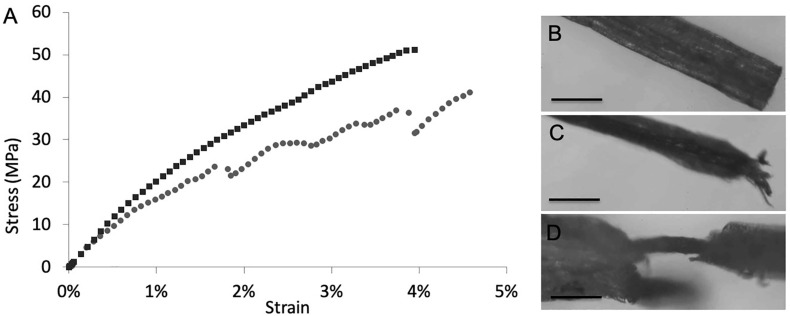
**(A)** Example stress-strain curves of untreated (black) and treated (gray) roots. After tensile tests, **(B)** characteristic fracture surface of untreated root samples showing a neat edge. Scale bar of 200 μm. **(C,D)** characteristic fracture surface of SWCNT-COOH treated roots showing **(C)** split filaments. Scale bar of 400 μm or, **(D)** plant vasculature channel filled by SWCNT-COOH no longer covered by the cellulosic layer. Scale bar of 100 μm.

Mechanical properties of the composite materials (Supplementary Figure S8) were derived through tensile tests of root samples. Considering the high variability that always characterizes biological materials, there are no significant differences between treated and untreated roots in terms of Young modulus, fracture strain, and strength, where the presence of SWCNT-COOH results to affect slightly the mechanical properties of the *A. thaliana* roots. As it may happen in composite materials, this behavior, as observed in Figure 4A, indicates that a phase separation occurs and SWCNT-COOH do not interact uniformly with the biological matrix, that thus cannot take advantage of their higher mechanical performance. The treated samples often present a sequence of load drops, which can be ascribed to inhomogeneity in the sample, with stiffer and softer regions that reduce both the load standing and elongation capability. Such hypothesis is supported by optical investigation of samples after mechanical tests. In fact, as shown in Figures 4B–D, the fracture surface of untreated root samples usually showed a neat edge (Figure 4B), while in the fractured surface of SWCNT-COOH treated roots it is possible to see the external part of the root broken while the inner channels containing SWCNT-COOH are still intact, owing to their higher mechanical resistance (Figures 4C,D).

## Discussion

For the biogenic incorporation of SWCNT-COOH into roots, we selected the plant *A. thaliana*, widely used as a model organism in plant biology. Initially, the toxicity of SWCNT-COOH toward the plant and the ability to incorporate SWCNT-COOH in the germination stage was verified. Water uptake is a critical step in seed germination. In fact, mature seeds are relatively dry and need a substantial amount of water to initiate cellular metabolism and growth. Imbibition is the physical process that marks the start of germination. The incorporation of SWCNT-COOH in the nascent plant may happen at that stage, as CNTs have been shown to penetrate the seed-coat and stimulate the seed germination due to more efficient water uptake induced by the CNT (Villagarcia et al., 2012). However, the germination of *A. thaliana* was not affected by the presence of SWCNT-COOH, suggesting that no incorporation of the SWCNT-COOH happened. The agar matrix present in the growth media entraps the SWCNTs-COOH (Supplementary Figure S3), and consequently, their effect on seed germination is limited.

Then, hydroponic culture was performed to study the effect of SWCNT-COOH on adult plants. Adult plants kept in deionized water become turgid due to a massive internalization of water caused by the difference in osmotic pressure (Munns et al., 2000; Schopfer, 2006). This phenomenon was exploited to favor the uptake of SWCNT-COOH. All the samples treated with SWCNT-COOH showed an increased mortality compared to the control, meaning that at this stage of the life cycle SWCNT-COOH have a toxic effect on plants, due to SWCNT-COOH incorporation. Counterintuitively, at the maximum concentration (10 μg/mL) the minimum toxic effect was observed. This occurs because at high concentrations aqueous dispersion of SWCNT-COOH associate into bundles, see Supplementary Figure S6 (Koh and Cheng, 2016), that are too big to be absorbed by the root. At the intermediate concentration of SWCNT-COOH (1 μg/mL) the maximum toxic effect was observed. At this concentration the SWCNT-COOH are well dispersed and can be internalized by the roots, causing a high mortality due to their toxic effect. Aggregation of the SWCNTs significantly affects their ability to interact with plants (Villagarcia et al., 2012). At the concentration of 0.1 μg/mL, the toxic effect of SWCNT-COOH was greatly reduced.

Based on the mortality test results, the roots treated with 1 μg/mL SWCNT-COOH were selected for further experiments, because when the maximum toxic effect is observed, a higher SWCNT-COOH incorporation is expected. After exposition to SWCNT-COOH, the roots of the survived plants became black. The obtained SWCNT-COOH/root composite was characterized.

Nanomaterials can be absorbed by primary and lateral roots or can be simply adsorbed on the surface of roots (Ma et al., 2010; Pérez-de-Luque, 2017). To discriminate between incorporation or surface adsorption, it is necessary to observe the inner vessels of the root (xylem and phloem), which can be accessible by slicing the root perpendicularly to its axis. The roots were sliced and analyzed with SEM and SECM.

Globally, non-treated roots showed a typical insulating behavior, while SWCNT-COOH treated samples behave as electric conductors, showing that the electrical properties of the conductive SWCNT-COOH are transferred to the insulating cellulose matrix, and demonstrating the fabrication of a biogenic composite material. The incorporation of the SWCNT-COOH influences also the macroscopic proprieties of the material, lowering the resistivity of the roots of 6 times. The increased conductivity observed in well-defined regions of the root, as showed by the overlap of SECM map and a SEM image (Figure 3A), corresponding to root vascular bundles can be ascribed to the incorporation of the SWCNT-COOH in the plant vessels, characterized in the section by a circular region (indicated by black arrows in Figure 3A). Plant vascular bundle includes xylem and phloem that transport water and mineral ions (xylem) and photosynthates (phloem) from and to the roots, respectively. The above experimental data suggests that SWCNT-COOH can enter into root xylem and assembly orienting along their main axis. These results are in agreement with previous studies, where carbon nanotubes were detected in root xylem, suggesting that CNTs penetrated the epidermal tissue and root cap to enter the root xylem (Zhai et al., 2015).

As described for other carbon nanomaterials (Villagarcia et al., 2012; Zhai et al., 2015) and nanoparticles (Wang et al., 2012; Ma et al., 2017; Sun et al., 2020), SWCNT-COOH translocation takes place along with the uptake of water, then the nanoparticles are transported through the xylem.

Localization of SWCNT-COOH in the vascular bundles is also supported by mechanical studies. In fact, incorporation of SWCNT-COOH affects slightly the mechanical properties of the *A. thaliana* roots. However, the stress-strain curves of SWCNT-COOH treated roots shows a sequence of load drops, typical of composite materials. This behavior is also supported by optical investigation of samples after tensile tests. In fact, the characteristic fracture surface of untreated root samples shows a neat edge, while the fracture surface of SWCNT-COOH treated roots shows split filaments. The local fracture of the external layer (more or less severe and visible from optical inspection) can thus be correlated to the load drops observed in the stress-strain curve.

## Conclusion

This work demonstrates for the first time that it is possible to create a new bionic material by growing *A. thaliana* in a medium containing SWCNT-COOH. SWCNT-COOH can enter the vascular bundles of *A. thaliana* roots localizing within xylem vessels. The conductive electrical properties of the SWCNT-COOH are transferred to the root matrix, which is intrinsically insulating. SWCNT-COOH are internalized and localized in the root’s xylem giving rise to both micro- and macro-scale conductivity of the composite. Stress-strain tests demonstrated the typical behavior of a composite, with stiffer and softer regions evidenced by a more resistant inner channel and a less resistant external layer. Even if the use of plants as bioreactors may introduce technological issues due to biological variability as repeatability of the synthetic process, yield for cycle, and downstream processing, all the observations confirm the successful entrapment of SWCNT-COOH by *A. thaliana* roots and demonstrate the fabrication of a “bionic composite,” posing the basis for the *in vivo* synthesis of new materials taking advantage of the living organism as a reactor.

## Data Availability Statement

The raw data supporting the conclusions of this article will be made available by the authors, without undue reservation.

## Author Contributions

GF, NMP, and MC contributed to the conception, design, and execution of this study. GM, MP, SR, LT, MD, FV, and FS executed the experiments described in this study. GF, NMP, and MC developed the analysis methodology. MC and GF drafted the manuscript with revisions contributed by all authors. All authors contributed to manuscript revision, and read and approved the submitted version.

## Conflict of Interest

The authors declare that the research was conducted in the absence of any commercial or financial relationships that could be construed as a potential conflict of interest.
