# Supplementary material for: A Plant Bioreactor for the Synthesis of Carbon Nanotube Bionic Nanocomposites
Source: Front Bioeng Biotechnol. 2020 Nov 5;8:560349. doi: 10.3389/fbioe.2020.560349 (PMC7676904; doi:10.3389/fbioe.2020.560349)
Supplement: Supplementary file 1 [file Table_1.docx]

Supplementary Material

A plant bioreactor for the synthesis of carbon nanotube bionic nanocomposites

# Giulia Magnabosco, Maria F. Pantano, Stefania Rapino, Matteo Di Giosia, Francesco Valle, Ludovic Taxis, Francesca Sparla, Giuseppe Falini, Nicola M. Pugno, and Matteo Calvaresi

Figure S1 pag. S2

Figure S2 S3

Figure S3 S4

Figure S4 S5

Figure S5 S6

Figure S6 S7

Figure S7 S8

Figure S8 S9


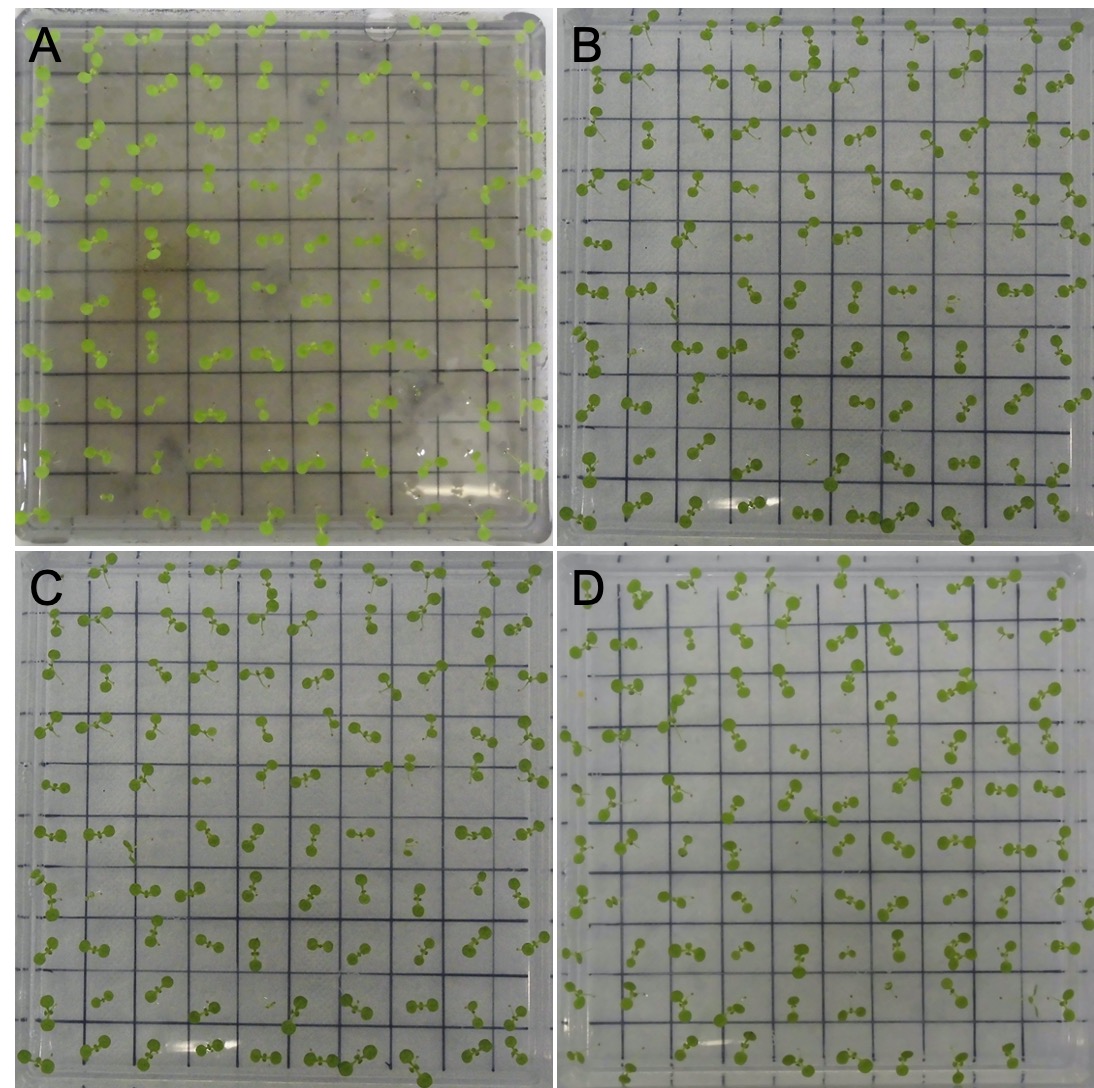


**Figure S1.** Germination dishes with SWCNT-COOH. (A) 10^-1^ mg/mL, (B) 10^-2^ mg/mL, (C) 10^-3^ µg/mL, (D) control.

**
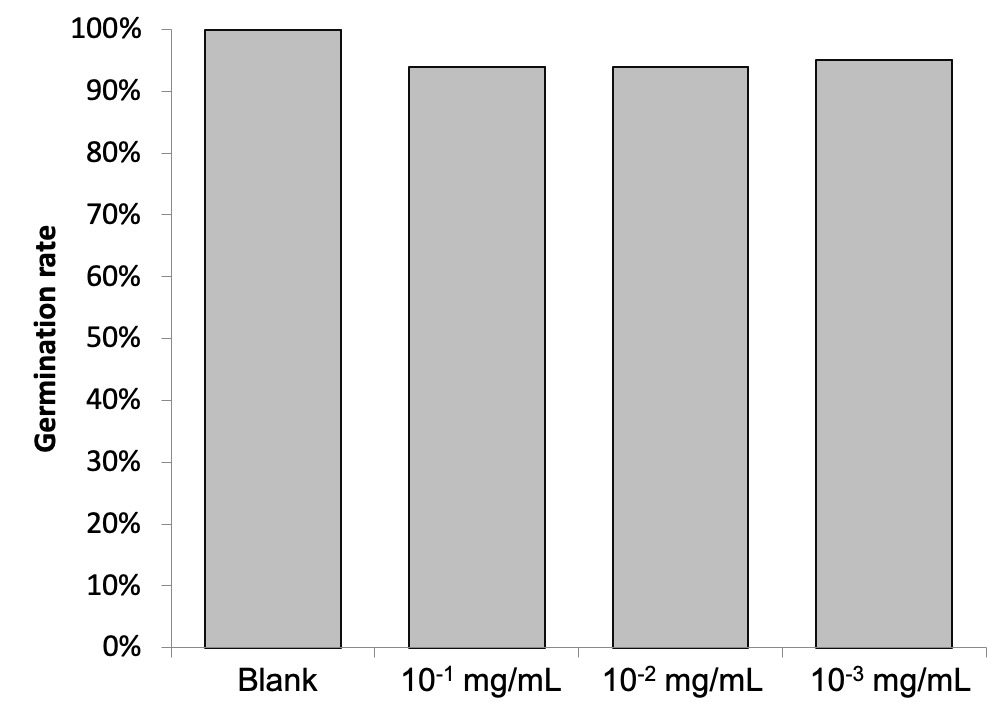
**

**Figure S2.** Germination rate of *A. thaliana* in the presence of SWCNT-COOH. n=100


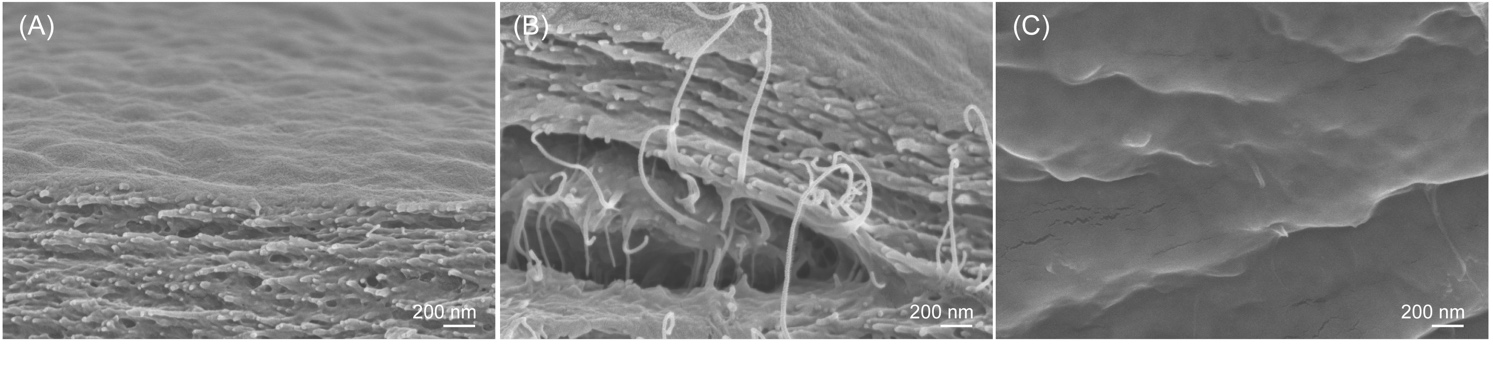


**Figure S3.** SEM images of a xerogel sample of (A) agar and (B) fractured and (C) intact SWCNT-COOH/agar (SWCNT-COOH = 10^-1^ mg/mL).


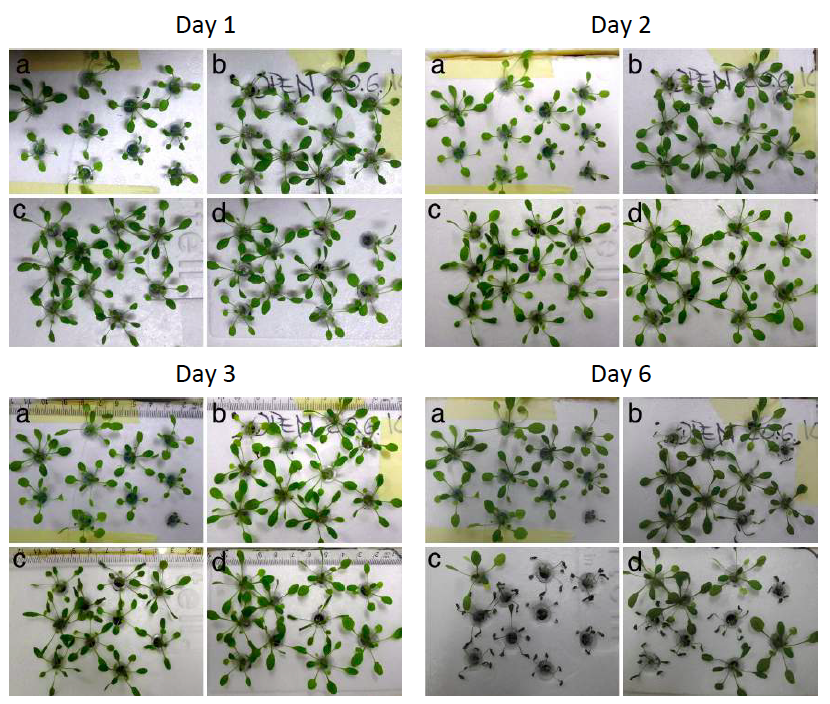


**Figure S4.** *A. thaliana* treated with SWCNT-COOH. (A) control, (B) 10 µg/mL, (C) 1 µg/mL, (d) 0.1 µg/mL.

**Figure S5.** Subsistence rate of *A. thaliana* treated with SWCNT-COOH after 6 days. n=10

DI water = Deionized water, control

**
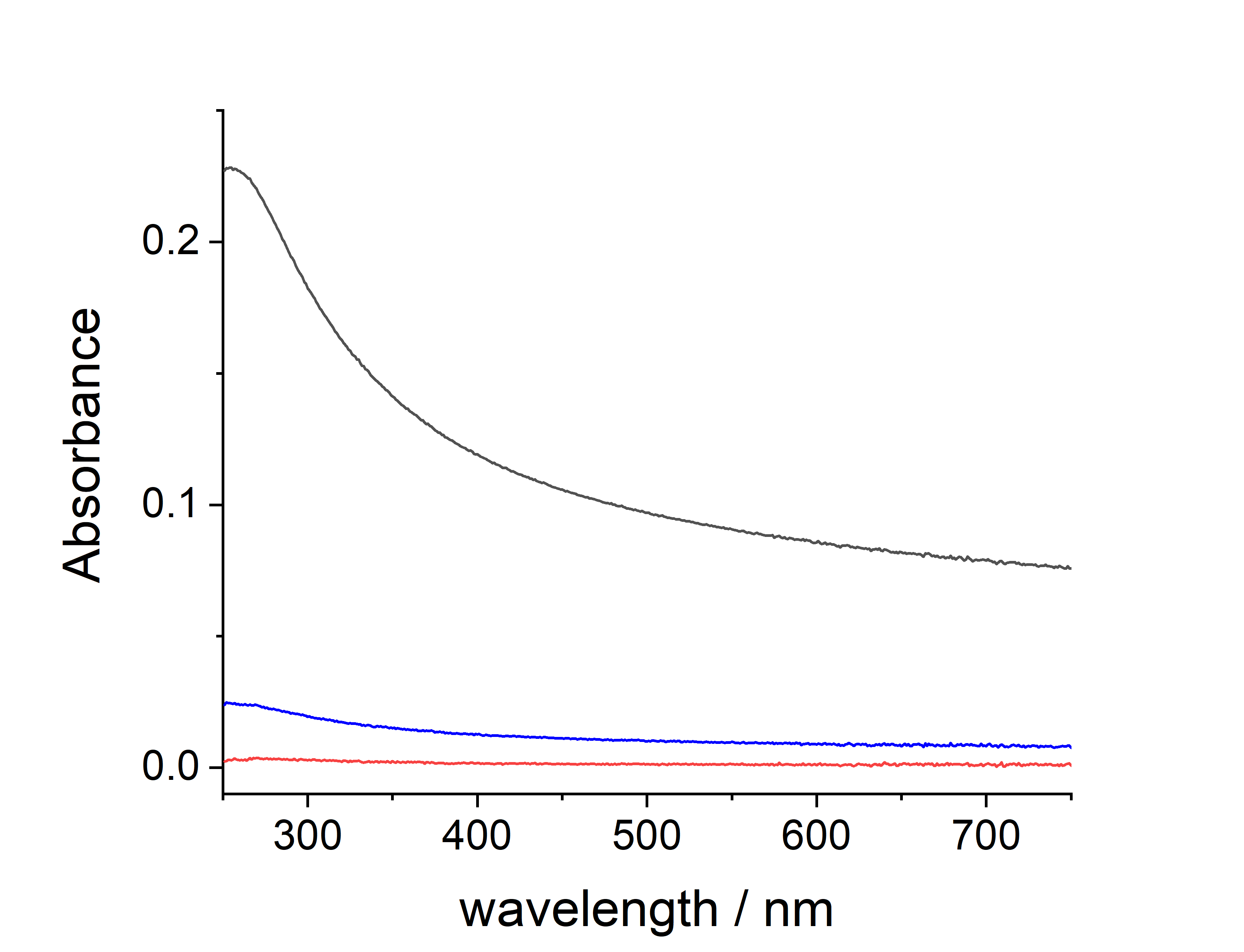
**

**Figure S6**. UV–vis absorbance spectra of 10 µg/mL SWCNT-COOH (black), 1 µg/mL SWCNT-COOH (cyan) and 0.1 SWCNT-COOH µg/mL (red).

**
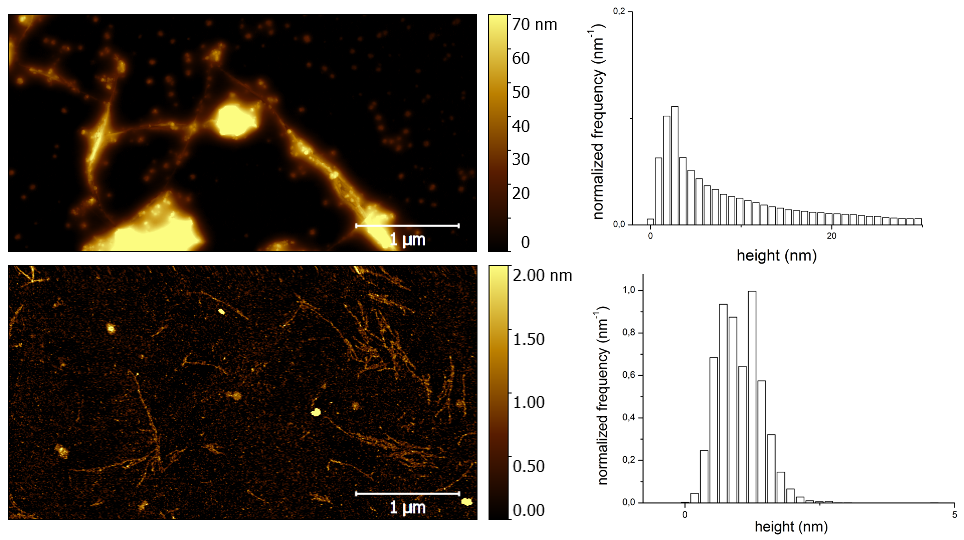
**

**Figure S7**. On the left. Morphological characterization of the dispersion state of 10 µg/mL SWCNT-COOH (A) and 1 µg/mL SWCNT-COOH (B). On the right. Height distribution analysis of the AFM images.


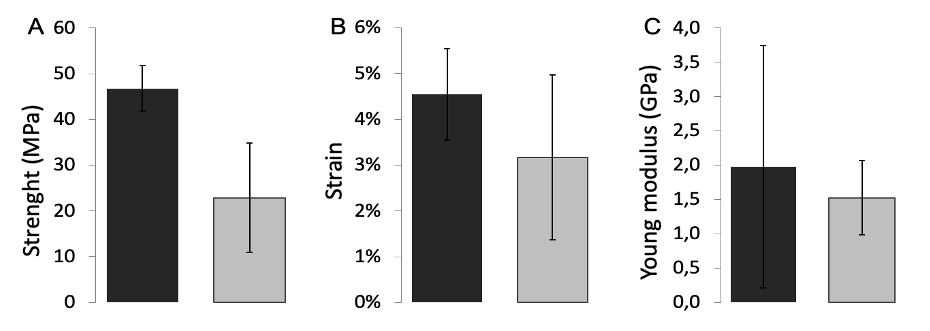


**Figure S8.** (A) Strength (p-value = 0.052), (B) fracture strain (p-value = 0.38) and (C) Young modulus (p-value = 0.15) of untreated (black) and SWCNT-COOH treated (grey) *A. thaliana* roots. Values obtained as average of six different measurements on six different roots.
